# Associations between serum sex steroid hormone metabolites and gastric cancer and precancerous lesions in men: A 11.8-year prospective study

**DOI:** 10.1515/jtim-2025-0041

**Published:** 2025-10-16

**Authors:** Jiayue Li, Wei Cui, Feifan He, Zhiyuan Fan, Liyan Xue, Wei Rao, Zongdan Wang, Zeming Wu, Jianhua Gu, Xinqing Li, Wenqiang Wei, Shaoming Wang

**Affiliations:** National Central Cancer Registry Office, National Cancer Center/National Clinical Research Center for Cancer/Cancer Hospital, Chinese Academy of Medical Sciences and Peking Union Medical College, Beijing, China; Department of Clinical Laboratory, State Key Laboratory of Molecular Oncology, National Cancer Center/National Clinical Research Center for Cancer/Cancer Hospital, Chinese Academy of Medical Sciences and Peking Union Medical College, Beijing, China; Department of Pathology, National Cancer Center/National Clinical Research Center for Cancer/Cancer Hospital, Chinese Academy of Medical Sciences and Peking Union Medical College, Beijing, China; iPhenome Biotechnology (Yun Pu Kang) Inc., Dalian, Liaoning Province, China; Department of Emergency Medicine, Qilu Hospital of Shandong University, Jinan, Shandong Province, China

**Keywords:** serum sex steroid hormone metabolites, gastric cancer, high-grade lesions, intestinal metaplasia, prospective cohort

## Abstract

**Background and objectives:**

Sex steroid hormones have been hypothesized to be associated with the risk of gastric cancer (GC); however, it has not been widely validated in prospective studies. We aimed to investigate the associations between sex steroid hormone metabolites and the risk of gastric cancer and precancerous lesions in a prospective cohort of Chinese men.

**Methods:**

Using liquid chromatography-tandem mass spectrometry (LC-MS/MS) and electrochemical luminescence immunoassay, we examined 20 sex steroid hormone metabolites and sex hormone-binding globulin (SHBG) in serum from 470 eligible men, including high-grade lesions or GC (*n* = 32), intestinal metaplasia (IM, *n* = 146), and 1:2 matched normal participants (*n* = 292) from 2007 to 2012. IM and normal participants were further followed up until December 2021, during which 32 new GC cases were identified with a median follow-up of 11.8 years. Associations between baseline sex steroid hormone metabolites and IM, high-grade lesions and gastric cancer were assessed using logistic regression, and associations between sex steroid hormone metabolites and incident GC risk were assessed using Cox proportional hazards regression in the prospective analysis.

**Results:**

In the cross-sectional analysis, androstenedione levels were potentially associated with IM risk and significantly associated with high-grade lesions or GC risk (OR_continuous_ = 2.45, 95% CI: 1.01–5.95). Higher concentrations of 17α-hydroxypregnenolone (OR_continuous_ = 2.35, 95% CI: 1.13–4.88), progesterone (OR_continuous_ = 2.68, 95% CI: 1.09–6.61), and estrone (OR_continuous_ = 5.36, 95% CI: 1.28–22.52) were also associated with an increased risk of high-grade lesions or GC. Furthermore, significant positive associations between GC risk and serum levels of SHBG (HR_continuous_ = 2.57, 95% CI: 1.04–6.36), epitestosterone (HR_continuous_ = 2.10, 95% CI: 1.07–4.15) and pregnenolone (HR_continuous_ = 1.30, 95% CI: 1.03–1.63) were identified in the follow-up study focusing on participants diagnosed as normal or IM. Notably, the subgroup analyses stratified by *H. pylori* status revealed similar associations between androstenedione, 17α-hydroxypregnenolone, progesterone, estrone, SHBG, pregnenolone, and GC risk.

**Conclusions:**

Several sex hormone metabolites were significantly associated with gastric cancer and its precancerous lesions, indicating a role for sex hormones in gastric carcinogenesis and potentially providing novel biomarkers for the identification of high-risk populations and risk prediction for GC.

## Introduction

Gastric cancer (GC) is the fifth most common malignancy globally, exhibiting striking geographic and ethnic disparities in its distribution. East Asian populations, particularly in China, bear the highest burden, with China alone accounting for 37.0% of global GC cases and 39.5% of GC-related deaths.^[1, 2, 3]^ This epidemiological pattern correlates strongly with *Helicobacter pylori* infection rates, a key driver of gastric carcinogenesis through chronic inflammatory processes. In China, the *H. pylori* prevalence reaches 49.6%,^[4]^ sustaining pathological progression from intestinal metaplasia (IM, a critical precancerous lesion) through dysplasia to invasive adenocarcinoma.^[5]^ Regional differences in the natural history of IM are also notable: while the annual progression rate from IM to GC is approximately 0.25% in Western Europe, it reaches 10% in East Asian populations.^[6,7]^ Reflecting this risk, China’s 2024 Guidelines for GC Screening and Early Diagnosis/Treatment recommend stratified endoscopic surveillance (at 1- to 3-year intervals) based on IM severity. These guidelines highlight the urgent need for early identification of IM in high-risk populations.

Another defining feature of GC burden is its marked sex disparity. In China, the age-standardized incidence rate of GC in 2022 was more than twice as high in men (19.47 per 100, 000) as in women (8.29 per 100, 000).^[8]^ Traditionally, this disparity has been attributed to differences in exposure to established risk factors such as smoking, alcohol use, and *Helicobacter pylori* (*H. pylori*) infection.^[9,10]^ However, accumulating evidence suggests that these factors alone cannot fully explain the observed sex differences.^[11,12]^ Sex hormones have been implicated in gastric carcinogenesis. This includes: (a) associations between reproductive factors and the risk of GC from results of epidemiological surveys;^[13,14]^ (b) associations between hormone replacement therapy and a lower GC risk,^[15]^ as well as anti-estrogen therapy and a higher GC risk in women;^[16]^ and (c) association between androgen deprivation therapy and a lower GC risk in men with prostate cancer.^[17]^ Experimental studies have also provided evidence to support this hypothesis, including the involvement of sex steroid hormones in the inflammatory process,^[18,19]^ and the expression of sex hormone receptors in GC tissue.^[20]^ In addition, a Mendelian randomization analysis using public Genome-Wide Association Study statistics showed a role of testosterone in the development of GC.^[21]^

Given this context, a growing number of studies have begun to explore associations between circulating sex hormone metabolites and GC risk. For example, a case-control study in a Mexican population found that higher concentrations of circulating dehydroepiandrosterone (DHEA) were associated with a lower risk of GC,^[22]^ while a prospective study in the UK reported that elevated sex hormone-binding globulin (SHBG) levels increased GC risk in men.^[23]^ Conversely, a nested case-control study conducted in the Chinese population reported no overall association between androgens and estrogens and GC risk.^[24]^ These inconsistencies may be attributed to differences in study design (*e.g*., case-control^[22]^ *vs*. prospective ^[23,24]^ study), population background (*e.g*., European and American ^[22,23]^ *vs*. East Asian^[24])^, and the sensitivity of laboratory methodologies (*e.g*., gas chromatography-mass spectrometry;^[22]^ chemiluminescent immunoassay;^[23]^ radioimmunoassays ^[24]^). Notably, most existing research has been conducted in Western populations and has focused primarily on GC, with limited investigation into East Asian populations or earlier precancerous stages such as IM. To address this gap, our study aims to evaluate the associations between serum sex steroid hormone metabolites and the risk of both GC and precancerous lesions, including IM, in a high-risk East Asian population.

We conducted this research in Linzhou, a rural county in China’s Henan Province, located in the Taihang Mountain area, which has among the highest GC incidence rates nationwide (140.5 per 100, 000 for cardia GC and 37.4 per 100, 000 for non-cardia GC), with local *H. pylori* prevalence ranging from 35.51% to 46.80%.^[4,25,26]^ A male screening cohort has been established in this region, enabling us to conduct a prospective study to investigate the role of serum sex steroid hormones in gastric carcinogenesis. The findings from this study are expected to provide novel biomarkers for the early detection of high-risk individuals and inform precision screening strategies for GC prevention in China and other high-burden regions.

## Materials and methods

### Selection and description of participants

A population-based upper gastrointestinal cancer screening cohort was established from 2007 to 2012 in Linzhou, China. Given the substantial differences in sex hormone levels between men and women, and the fact that women experience significant hormonal fluctuations before and after menopause while men’s hormone levels decline gradually with age and are unaffected by reproductive cycles, we selected men as study participants. This approach ensures hormonal stability and is consistent with the design of previous related studies. After a baseline questionnaire interview and physical examination, a total of 558 men were enrolled and 10 mL of fasting blood was collected from each participant who had fasted for at least 12 h prior to the physical examination. Serum samples were immediately separated, aliquoted, and stored frozen at -70 ℃ until they were prepared for the laboratory analysis.

The endoscopic examination and biopsy procedures were described in detail in a previous study.^[27]^ Specifically, the participants received a local anesthetic (5 mL of 1% lidocaine solution) orally 5 min before endoscope insertion, followed by a complete visual examination of the esophagus and stomach. Then, the stomach was sprayed with 0.2% indigo carmine, which stains the gastric fundic gland mucosa light red and imparts dull yellow to the pyloric gland mucosa. In areas of abnormal gastric mucosa, the dye demonstrates abnormal deposition, resulting in accentuated staining. Next, all areas with accentuated staining > 5 mm in diameter were biopsied, with the number of specimens collected (range 1–3) depending on lesion dimensions (5–19 mm, 1 biopsy; 20–39 mm, 2 biopsies; and ≥40 mm, 3 biopsies). Biopsy specimens underwent standard processing including 10% buffered formalin fixation, paraffin embedding, sectioning at 5-μm, and hematoxylin-eosin (HE) staining. All sides were independently evaluated by two well-trained pathologists, with discordant cases reviewed jointly to reach diagnostic consensus. Finally, the histological criteria strictly adhered to the 2020 edition of Chinese Technical Guidelines for Screening, Early Diagnosis, and Treatment of Upper Gastrointestinal Cancers.^[28]^

**Figure 1:**
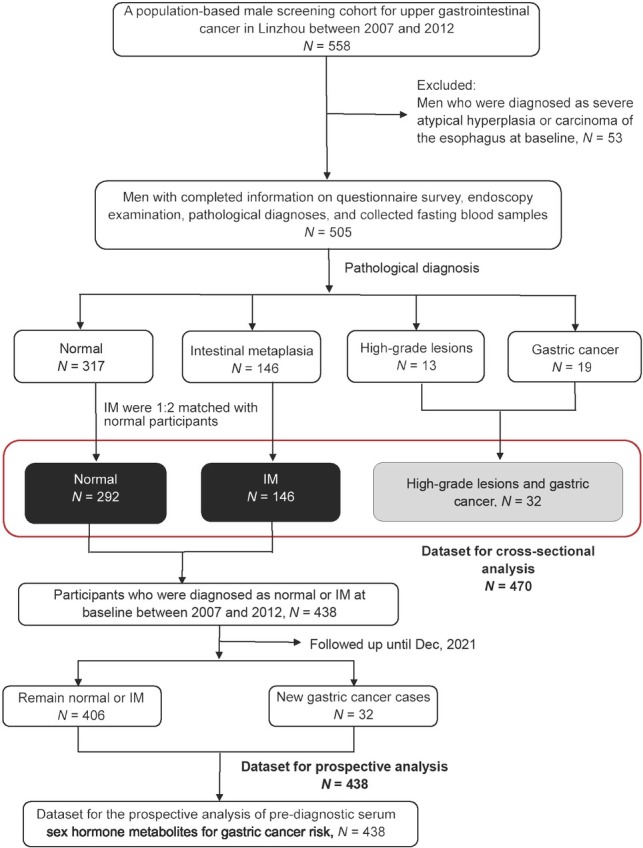
Flowchart of the study for diagnostic analysis and prospective analysis. IM, intestinal metaplasia; Gastric cancer includes non-cardia gastric cancer and cardia gastric cancer.

We excluded men who were diagnosed as severe atypical hyperplasia or carcinoma of the esophagus at baseline (*n* = 53) (Figure 1). Among the 505 eligible men, there were 32 participants diagnosed with GC (*n* = 19) or high-grade lesions (*n* = 13), 146 diagnosed with IM, and 317 normal participants. We randomly matched the IM cases with normal participants by age and village with a ratio of 1:2, and finally included 32 high-grade lesions and GC cases, 146 IM cases, and 292 normal participants in the cross-sectional analytical dataset (*n* = 470). From 2007 to 2021, the village health workers checked vital status and the occurrence of incident cancers and ascertained causes of deaths for all participants by monthly home visits, supplemented by quarterly crosschecks of the data in the Linzhou Cancer and Death Registries. New GC cases and all causes of death were reviewed by a panel of professional experts from the Cancer Hospital, Chinese Academy of Medical Sciences in Beijing, China. Diagnostic materials used in these expert reviews included medical records, pathology and cytology slides, biochemical results, X-rays, ultrasound, endoscopy, and surgical reports. The International Classification of Diseases for Oncology, Third Revision code C16.0 was used to define CGC, and NCGC was defined with topography code C16.1–C16.9.^[29]^ Primary causes of death were classified by the International Classification of Diseases-10.^[30]^ By December 2021, a total of 32 new GC cases were diagnosed in the prospective analytical dataset (*n* = 438, including 146 IM and 292 normal participants).

### Data collection

All participants underwent a physical examination at local hospitals and completed a standardized questionnaire according to the national upper gastrointestinal cancer screening guideline.^[29]^ The questionnaire included items on demographics (age, sex and residence location), height, weight, smoking status (regular cigarette or pipe use for at least 6 months), alcohol consumption (any alcohol consumption in the past 12 months), history of upper gastrointestinal disease (*i.e*., ulcer, esophagitis, gastroenteritis, and hepatitis), and family history of cancer (at least one first-degree relative with cancer).

### Laboratory analysis

*H. pylori* infection was measured by immunoblot assay at the laboratory of Linzhou Cancer Hospital. SHBG was tested with electrochemical luminescence immunoassay at the laboratory of the Cancer Hospital, Chinese Academy of Medical Sciences. Sex steroid hormone metabolites were assessed by liquid chromatography-tandem mass spectrometry (LC-MS/MS) system at the Integrated Chinese and Western Medicine Laboratory of Dalian Medical University. A total of 20 sex steroid hormone metabolites were quantified, including testosterone, 16 α-hydroxytestosterone, androstenedione, 17α-hydroxyprogesterone, 17α-hydroxypregnenolone, progesterone, estrone, epitestosterone, DHEA, pregnenolone, dihydrotestosterone, etiocholanolone, 17-epiestriol, estradiol, 2-methoxyestrone, 2-methoxyestradiol, 16-epiestriol, androsterone, 11-oxoetiocholanolone, and 6β-hydroxytestosterone. Quality control (QC) samples were prepared by evenly mixing aliquots of randomly assigned samples and were inserted into the analytical sequence. A total of 35 QC samples were evenly distributed on each solid phase extraction plate and analyzed by LC-MS/MS. The merits of the analytical performance, including reproducibility, *i.e*., coefficients of variance (CVs) of the concentration determinations in these QC samples, and sensitivity of the LC-MS/MS method (limits of detection (LODs), limits of quantification (LOQs)), were reported in the Supplementary Materials. In these QC samples, CVs were less than 46% (range = 1.14%–45.98%), LODs were less than 5 μg/L (range = 0.25–5 μg/L), and LOQs were less than 5 μg/L (range = 0.25–5 μg/L).

### Statistics

Baseline characteristics and the serum sex steroid hormone metabolites levels of participants were presented as median or percentage between normal and case groups. The Wilcoxon-Mann-Whitney test and chi-squared test were used to compare the statistical differences between groups for continuous and categorical variables, respectively. As the continuous sex hormone values were right-skewed, these values were logarithmically transformed to follow a normal distribution. In addition, sex steroid hormone metabolites were categorized into quartiles, based on the distributions among the normal participants at baseline. Tests for linear trend were performed on the basis of the quartiles.

In the cross-sectional analysis, conditional logistic regression, based on the age-matched IM-normal pairs, was used to estimate the odds ratios (ORs) and 95% confidence intervals (CIs) for associations between sex steroid hormone metabolites and IM risk. Binary logistic regression was used to evaluate ORs and 95% CIs for associations between sex steroid hormone metabolites and high-grade lesions or GC risk. In the prospective analysis, Cox proportional hazards regression was used to evaluate the hazard ratios (HRs) and 95% CIs for associations between sex steroid hormone metabolites and GC risk. The proportional hazards assumption was assessed using Schoenfeld’ residuals and Kolmogorov-type supremum analysis, with evidence of equiproportionality being detected. Follow-up time in years was used as the underlying time metric and was calculated from baseline to the date of diagnosis of GC, the date of death, or the end of the follow-up period (December 31, 2021), whichever occurred first. Multivariable models were adjusted for age, body mass index (BMI), smoking status, alcohol consumption, history of upper gastrointestinal disease, family history of cancer, and *H. pylori* CagA infection. Additionally, participants were stratified by the *H. pylori* CagA infection status for the sensitivity analysis to test the effects of *H. pylori* infection on associations between sex steroid hormone metabolites and GC and precancerous lesions.

In addition to individual sex hormones, we also calculated the following parameters using the aforementioned analytical approaches: parent estrogens (the sum of estrone and estradiol), testosterone: parent estrogens ratio, testosterone: estradiol ratio, and androstenedione: estrone ratio. Statistical significance was defined as *P* < 0.05, and all *P* values were two-tailed. Analyses were performed with SAS, version 9.4 (SAS Institute, Inc., Cary, NC) and R 4.3.2 (R Foundation for Statistical Computing, Vienna, Austria).

## Results

### Baseline characteristics and levels of serum sex hormone metabolites

Demographic characteristics, distributions of risk factors, and levels of serum sex hormone metabolites for participants with different baseline pathological diagnoses are summarized in Table 1. A total of 470 participants were enrolled in the cross-sectional analysis. Compared to normal participants, those with IM were more likely to be infected with *H. pylori* (65.1% *vs*. 52.1%, *P =* 0.01). High-grade lesions or GC patients were older than normal participants (73.5 years *vs*. 68.0 years, *P* < 0.01), and generally exhibited higher median concentrations of sex hormones compared to both IM patients and normal participants.

**Table 1:** Baseline characteristics and levels of serum sex hormone metabolites in subjects with different pathological diagnoses

	Normal participants (*N* = 292)	Intestinal metaplasia participants (*N* = 146)	*P* value	High-grade lesions or gastric cancer participants (*N* = 32)	*P* value
Age (years)					
Median [Q1, Q3]	68.0 [62.0, 73.0]	68.0 [62.3, 74.0]	0.38	73.5 [70.0, 78.3]	<0.01
BMI (Kg/m2)					
Median [Q1, Q3]	22.5 [20.8, 24.4]	22.2 [20.7, 24.2]	0.18	21.7 [20.5, 23.5]	0.16
Smoking (*n*, %)					
No	156 (53.4%)	84 (57.5%)	0.48	12 (37.5%)	0.13
Yes	136 (46.6%)	62 (42.5%)		20 (62.5%)	
Drinking (*n*, %)					
No	245 (83.9%)	126 (86.3%)	0.61	26 (81.3%)	0.89
Yes	47 (16.1%)	20 (13.7%)		6 (18.8%)	
History of upper gastrointestinal disease (*n*, %)					
No	240 (82.2%)	130 (89.0%)	0.08	28 (87.5%)	0.61
Yes	52 (17.8%)	16 (11.0%)		4 (12.5%)	
Family history of cancer (*n*, %)					
No	156 (53.4%)	77 (52.7%)	0.97	20 (62.5%)	0.43
Yes	136 (46.6%)	69 (47.3%)		12 (37.5%)	
*H.pylori* CagA* (*n*, %)					
No	140 (47.9%)	51 (34.9%)	0.01	13 (40.6%)	0.55
Yes	152 (52.1%)	95 (65.1%)		19 (59.4%)	
Sex hormone binding globulin (ng/L)					
Median [Q1, Q3]	69.1 [51.6, 94.5]	73.4 [52.1, 102.0]	0.21	87.8 [66.9, 107.0]	0.01
Testosterone (ng/L)					
Median [Q1, Q3]	6340 [5100, 7800]	6710 [5140, 8120]	0.17	7340 [5270, 9130]	0.13
16α-Hydroxytestosterone (ng/L)					
Median [Q1, Q3]	1460 [1110, 1870]	1520 [1110, 2010]	0.40	1430 [1120, 1750]	0.76
Androstenedione (ng/L)					
Median [Q1, Q3]	1250 [946, 1610]	1230 [1020, 1590]	0.52	1710 [1120, 2380]	<0.01
17α-Hydroxyprogesterone (ng/L)					
Median [Q1, Q3]	994 [784, 1250]	958 [744, 1230]	0.26	1210 [929, 1550]	0.04
17α-Hydroxypregnenolone (ng/L)					
Median [Q1, Q3]	3490 [2440, 5040]	3320 [2510, 4560]	0.49	4670 [2750, 6590]	0.11
Progesterone (ng/L)					
Median [Q1, Q3]	59.2 [47.4, 73.6]	56.6 [45.6, 68.7]	0.16	69.7 [49.1, 99.6]	0.07
Estrone (ng/L)					
Median [Q1, Q3]	63.5 [53.6, 75.2]	64.5 [54.4, 78.6]	0.28	79.5 [61.1, 93.1]	<0.01
Epitestosterone (ng/L)					
Median [Q1, Q3]	109.0 [68.7, 152.0]	109.0 [75.3, 161.0]	0.45	149.0 [91.2, 237.0]	0.02
Dehydroepiandrosterone (ng/L)					
Median [Q1, Q3]	1450 [992, 2130]	1430 [1000, 1960]	0.80	1370 [1040, 1920]	0.70
Pregnenolone (ng/L)					
Median [Q1, Q3]	519 [161, 788]	531 [185, 794]	0.83	665 [125, 1050]	0.28
Dihydrotestosterone (ng/L)					
Median [Q1, Q3]	559 [443, 760]	590 [450, 799]	0.38	618 [476, 786]	0.34
Etiocholanolone (ng/L)					
Median [Q1, Q3]	201 [152, 264]	214 [158, 282]	0.33	215 [159, 257]	0.97
17-Epiestriol (ng/L)					
Median [Q1, Q3]	14.9 [11.5, 19.3]	14.1 [10.6, 18.6]	0.18	13.0 [10.3, 18.3]	0.17
Estradiol (ng/L)					
Median [Q1, Q3]	36.9 [25.3, 47.1]	39.5 [25.5, 52.5]	0.26	36.1 [31.2, 43.0]	0.89
2-Methoxyestrone (ng/L)					
Median [Q1, Q3]	18.0 [13.6, 23.7]	17.7 [13.5, 23.3]	0.84	20.1 [15.4, 31.5]	0.16
2-Methoxyestradiol (ng/L)					
Median [Q1, Q3]	12.7 [9.2, 19.2]	12.2 [9.2, 19.2]	0.88	12.5 [8.2, 20.1]	0.97
16-Epiestriol (ng/L)					
Median [Q1, Q3]	37.1 [21.0, 54.6]	33.1 [17.1, 48.3]	0.14	31.5 [16.3, 54.1]	0.40
Androsterone (ng/L)					
Median [Q1, Q3]	356 [266, 462]	364 [288, 493]	0.29	343 [284, 442]	0.89
11-Oxoetiocholanolone (ng/L)					
Median [Q1, Q3]	58.2 [26.2, 142.0]	48.4 [23.8, 120.0]	0.25	76.8 [33.5, 147.0]	0.35
6β-Hydroxytestosterone (ng/L)					
Median [Q1, Q3]	65.6 [33.8, 136.0]	70.5 [39.0, 150.0]	0.26	86.6 [29.2, 169.0]	0.71
Parent estrogens (ng/L)					
Median [Q1, Q3]	102.0 [82.4, 119.0]	105.0 [85.3, 125.0]	0.22	112.0 [96.0, 136.0]	0.02
Testosterone: parent estrogens ratio					
Median [Q1, Q3]	62.2 [50.8, 81.5]	65.7 [48.5, 87.3]	0.55	65.6 [49.3, 84.7]	0.84
Testosterone: estradiol ratio					
Median [Q1, Q3]	176 [132, 250]	179 [122, 262]	0.83	194 [154, 272]	0.31
Androstenedione: estrone ratio					
Median [Q1, Q3]	19.1 [15.5, 24.6]	20.0 [15.9, 25.9]	0.49	22.4 [17.3, 29.8]	0.10

BMI: body mass index. *H.pylori CagA is one of the antibodies against *Helicobacter pylori*.

### Cross-sectional analysis results

**Figure 2:**
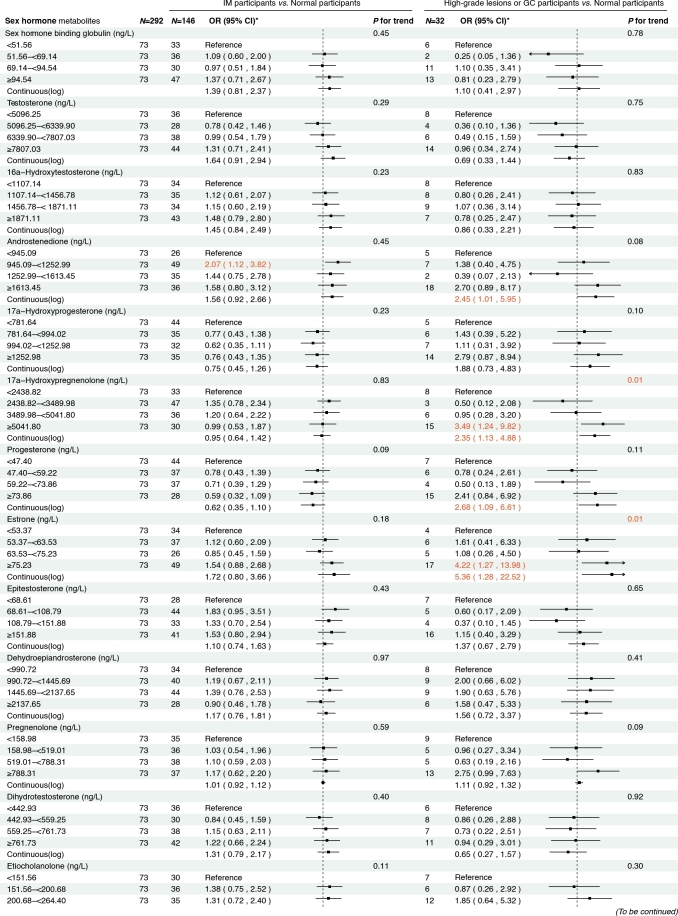

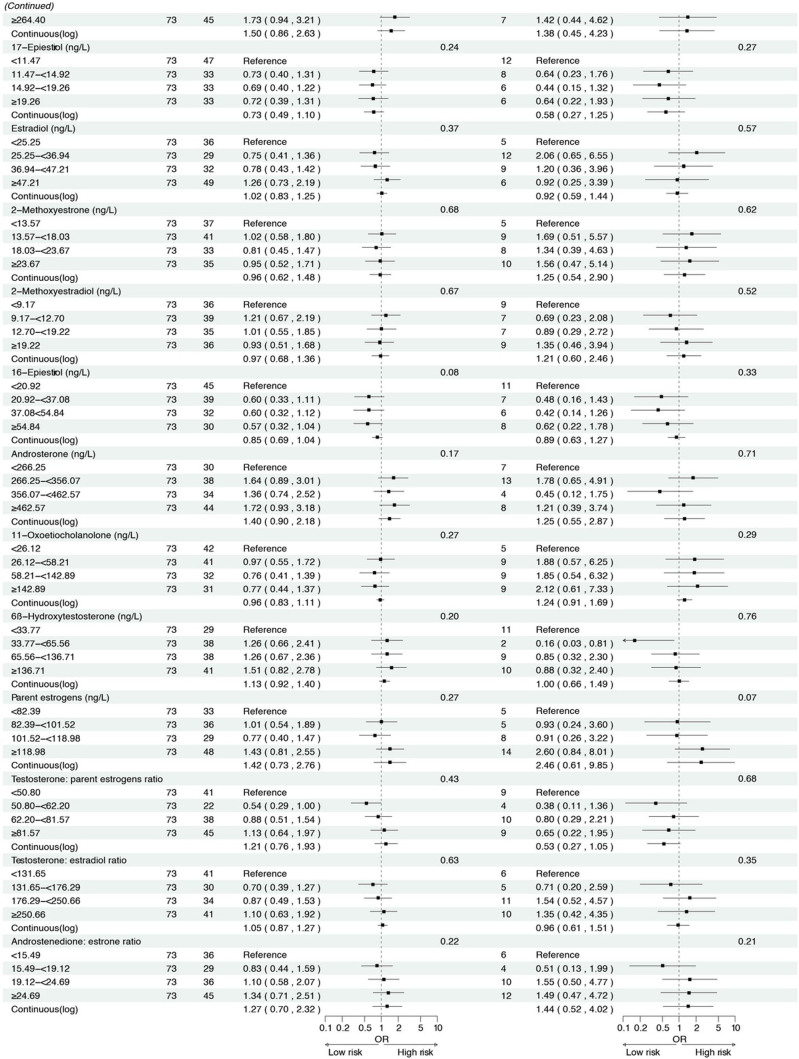
Associations between sex hormone metabolites and intestinal metaplasia and high-grade lesions or gastric cancer risk. *: Adjusted for age, body mass index, smoking, drinking, history of upper gastrointestinal disease, family history of cancer, *H.pylori* CagA. IM, intestinal metaplasia; GC, gastric cancer.

Figure 2 presents the associations between serum sex steroid hormone metabolites and the risk of IM and high-grade lesions or GC in the cross-sectional analysis. We found that higher concentrations of androstenedione were associated with an increased risk of high-grade lesions or GC (OR_continuous_= 2.45, 95% CI: 1.01–5.95), and a similar association was observed between androstenedione and IM (OR_Q2_ *vs*. _Q1_ = 2.07, 95% CI: 1.12–3.82). Meanwhile, higher concentrations of 17α-hydroxypregnenolone (OR_continuous_= 2.35, 95% CI: 1.13–4.88) and estrone (OR_continuous_ = 5.36, 95% CI: 1.28–22.52) were associated with an increased risk of high-grade lesions or GC, with consistent results when analyzing quartiles of 17α-hydroxypregnenolone (OR_Q4_ *vs*. _Q1_ = 3.49, 95% CI: 1.24–9.82, *P*_trend_ = 0.01) and estrone (OR_Q4_ *vs*. _Q1_ = 4.22, 95% CI: 1.27–13.98, *P*_trend_ = 0.01). Additionally, higher concentrations of progesterone (OR_continuous_ = 2.68, 95% CI: 1.09–6.61) were also associated with an increased risk of high-grade lesions or GC in participants.

### Prospective analysis results

**Figure 3:**
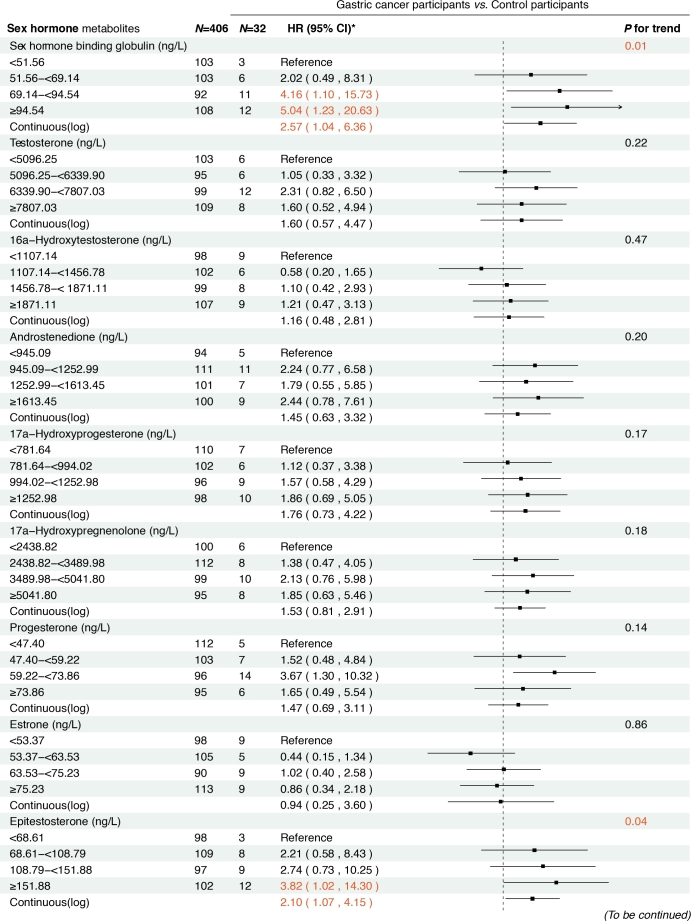

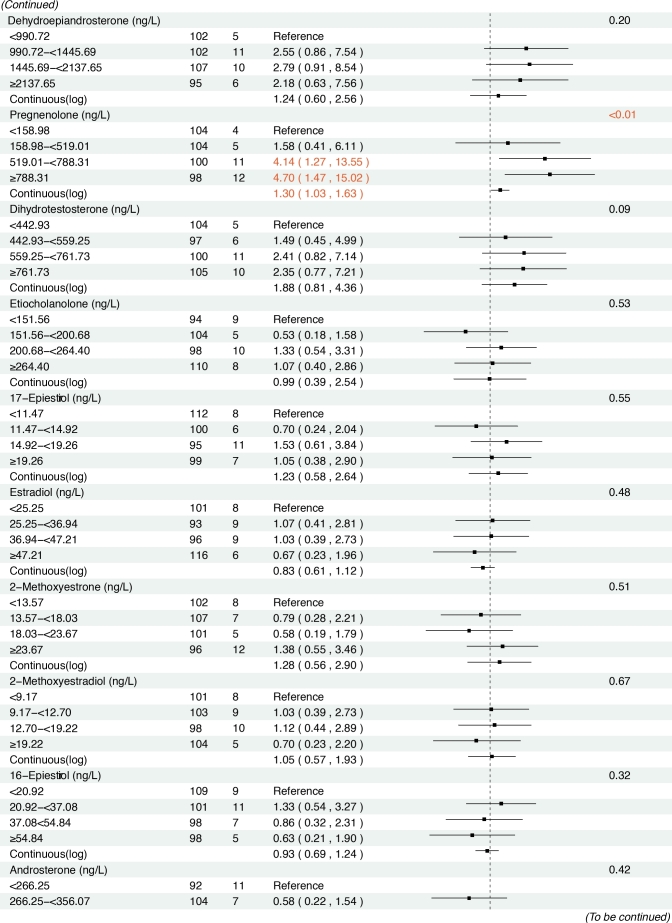

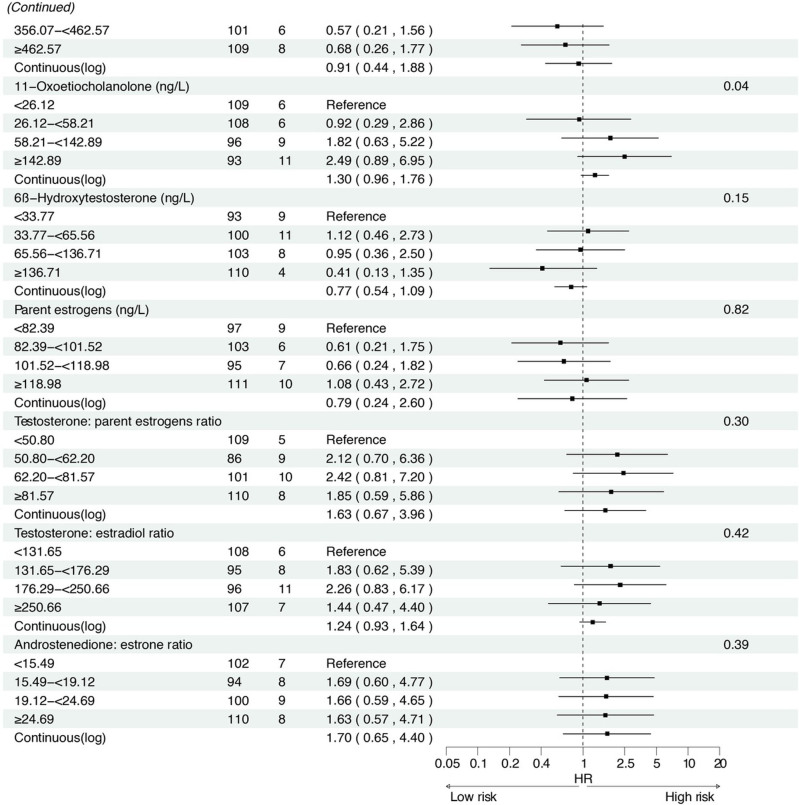
Associations between baseline sex hormone metabolites and incident gastric cancer risk from 2007 to 2021. *: Adjusted for age, body mass index, smoking, drinking, history of upper gastrointestinal disease, family history of cancer, *H.pylori* CagA.

In the prospective cohort of 438 participants diagnosed with either normal or IM at baseline, we found positive associations between several serum sex steroid hormone metabolites and GC risk during a median follow-up of 11.8 years (IQR: 9.7–13.7 years). Specifically, statistically significant associations were observed between higher levels of SHBG (HR_continuous_ = 2.57, 95% CI: 1.04–6.36), epitestosterone (HR_continuous_ = 2.10, 95% CI: 1.07–4.15), and pregnenolone (HR_continuous_= 1.30, 95% CI: 1.03–1.63) and incident GC risk (Figure 3). The results remained consistent when analyzing the quartiles of SHBG (HR_Q4_ *vs*. _Q1_ = 5.04, 95% CI: 1.23–20.63, *P*_trend_ = 0.01), epitestosterone (HR_Q4_ *vs*. _Q1_ = 3.82, 95% CI: 1.02–14.30, *P*_trend_ = 0.04), and pregnenolone (HR_Q4_ *vs*. _Q1_ = 4.70, 95% CI: 1.47–15.02, *P*_trend_ < 0.01).

### Sensitivity analysis

**Figure 4:**
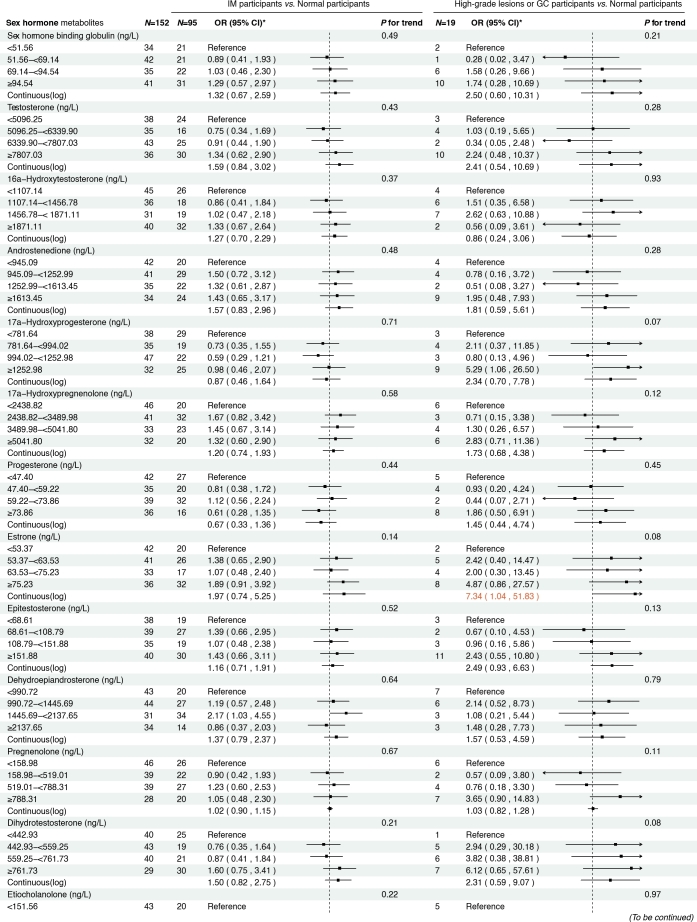

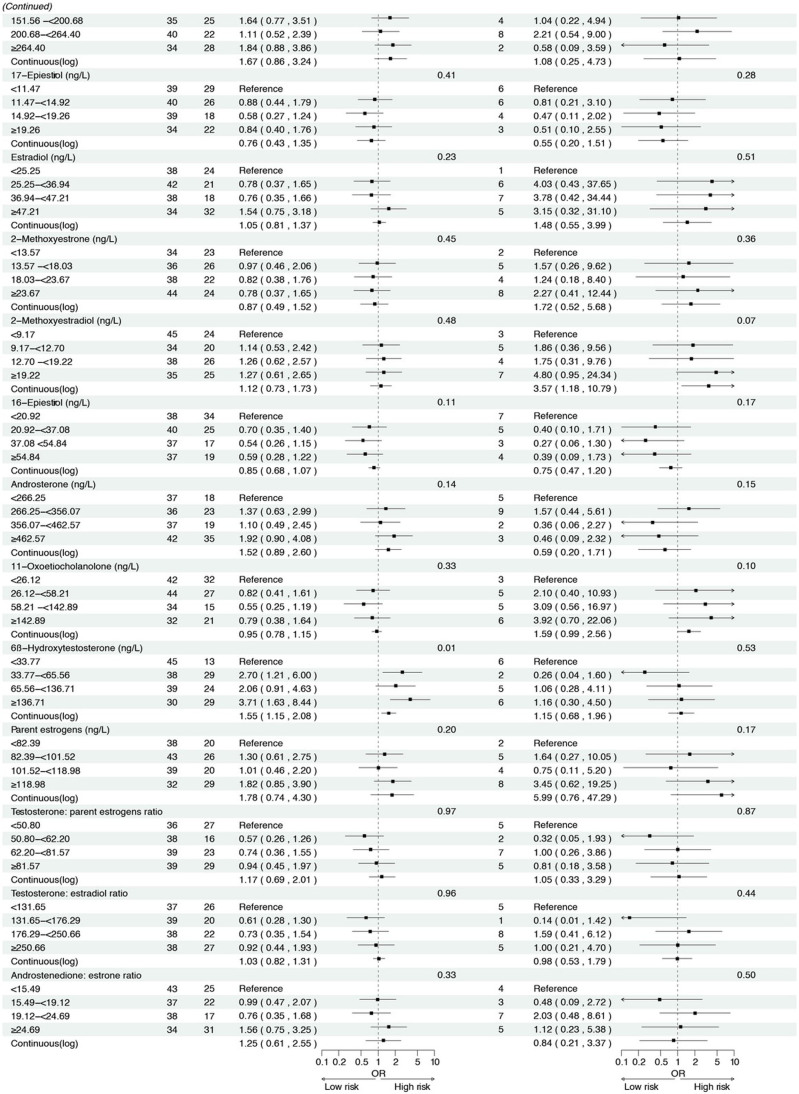
Associations between sex hormone metabolites and gastric cancer and its precursors among H. pylori CagA-positive participants. *: Adjusted for age, body mass index, smoking, drinking, history of upper gastrointestinal disease, family history of cancer. IM, intestinal metaplasia; GC, gastric cancer.

**Figure 5:**
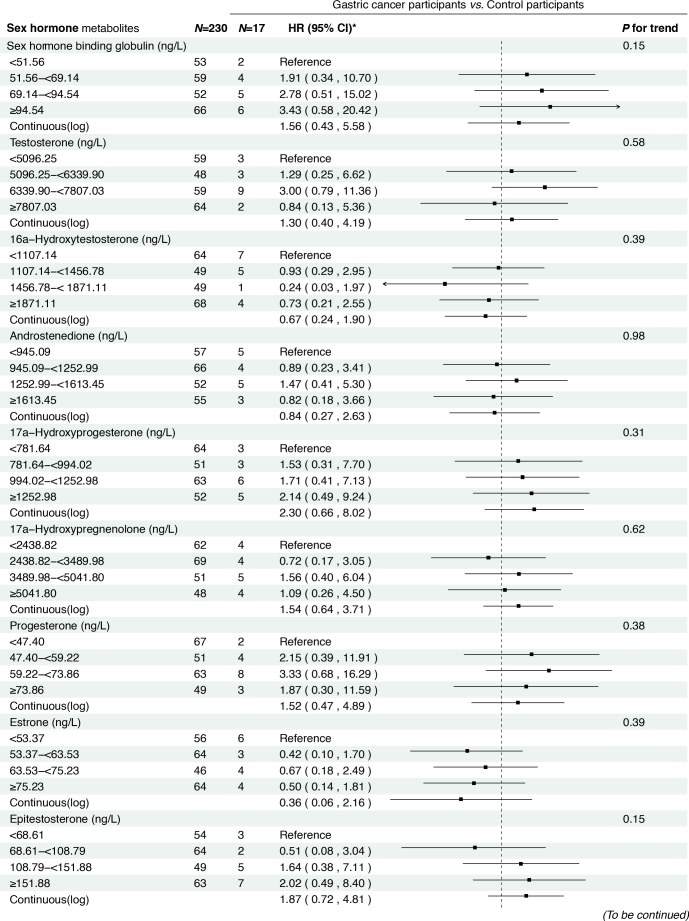

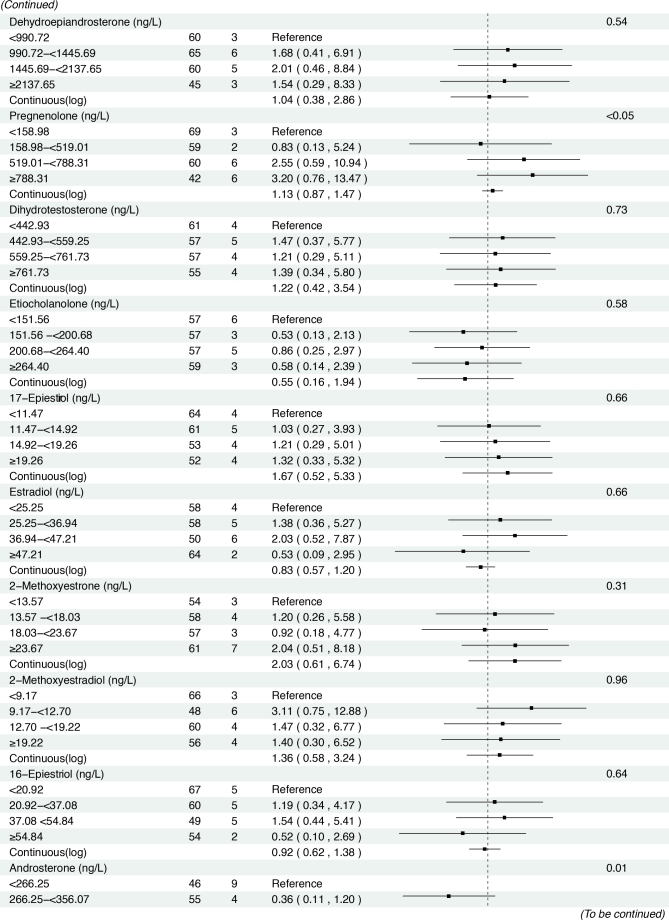

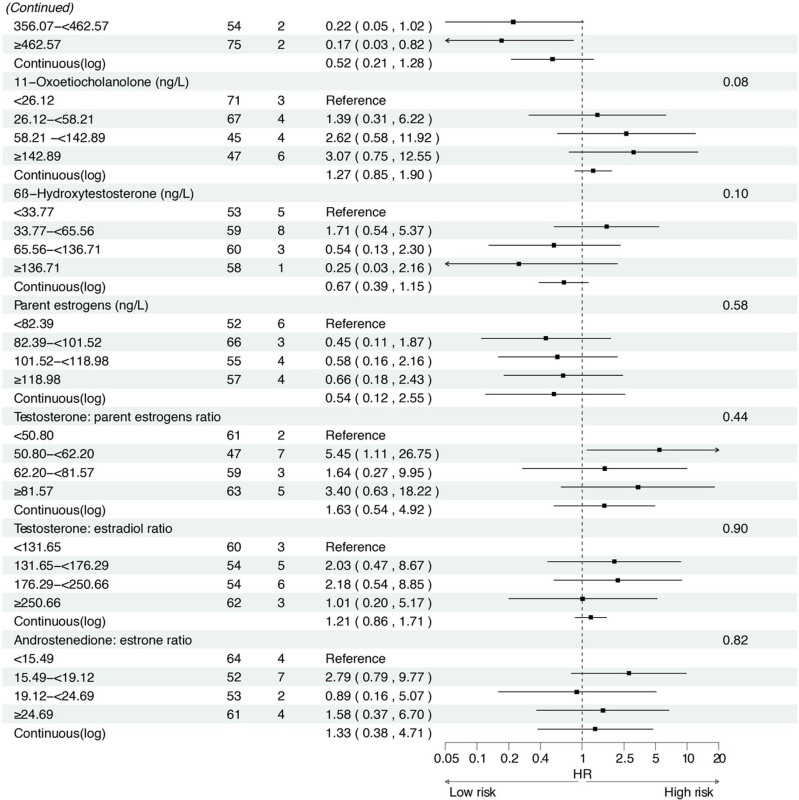
Associations between baseline sex hormone metabolites and incident gastric cancer risk among H. pylori CagA-positive participants. *: Adjusted for age, body mass index, smoking, drinking, history of upper gastrointestinal disease, family history of cancer.

To further explore the potential modifying effect of *H. pylori* infection, we stratified the analysis by *H. pylori* CagA serostatus. Consistent associations between sex steroid hormone metabolites and the risk of GC or its precursors were observed in both the overall dataset and the stratified subgroups. Among *H. pylori* CagA-positive participants, higher levels of estrone were significantly associated with high-grade lesions or GC risk (Figure 4–5). Meanwhile, in *H. pylori* CagA-negative participants, increased levels of androstenedione, 17α-hydroxypregnenolone, and progesterone were associated with the risk of high-grade lesions or GC, and both SHBG and pregnenolone exhibited positive associations with GC risk (Supplementary Figure S1–2). However, no statistically significant associations were observed between epitestosterone levels and the risk of GC and precancerous lesions in subgroup analyses, possibly reflecting limited statistical power due to small sample sizes within each stratum.

## Discussion

In this prospective cohort study of Chinese men, elevated serum concentrations of androstenedione, 17α-hydroxypregnenolone, progesterone and estrone were significantly associated with an increased risk of high-grade lesions or GC. During a median follow-up of 11.8 years, positive associations with incident GC risk were also observed for SHBG, epitestosterone and pregnenolone.

Notably, with the exception of epitestosterone, these associations remained robust across analyses stratified by *H. pylori* serostatus. Collectively, these findings underscore the potential contributory role for sex hormone metabolites in the gastric carcinogenesis.

To our knowledge, this is the first population-based study to explore associations between circulating sex hormones and the risk of GC and its precancerous lesions, including high-grade lesions and IM. In cross-sectional analyses, elevated androstenedione levels were associated with an increased risk of IM, with even stronger associations observed for high-grade lesions or GC (OR = 2.45). Although evidence linking androstenedione to IM is limited, with only one animal study implicating testosterone,^[31]^ the metabolic interconversion between testosterone and androstenedione,^[32]^ together with the observed progression risk, supports androstenedione as a promising biomarker in early gastric carcinogenesis. Notably, this association was markedly stronger in *H. pylori* CagA-negative individuals (OR = 4.84), suggesting a potentially distinct carcinogenic pathway independent of *H. pylori* infection.

Previous mechanistic studies have reported that androgens, including androstenedione, exert their biological effects through the androgen receptor (AR), which has been linked to increased GC risk, *H. pylori*-negative status, and immune evasion, such as CD 8^+^ T cell exhaustion.^[33,34]^ These findings support the hypothesis that the androgen-AR axis plays a key role in GC development, although further mechanistic validation is needed. In addition, higher levels of estrone, a precursor metabolized to estradiol, were also significantly associated with an increased risk of high-grade gastric lesions or GC (OR _continuous_ = 5.36). Mechanistically, estradiol has been shown to promote epithelial–mesenchymal transition in GC cells, an effect further potentiated by coinfection with CagA-positive *H. pylori*,^[35]^ which aligns with our subgroup analyses demonstrating stronger associations between estrone and high-grade lesions or GC in CagA-positive individuals (OR _continuous_ = 7.34). These findings suggest a synergistic interplay between sex hormones and *H. pylori* infection in gastric carcinogenesis.

Progesterone exerts its effects through the progesterone receptor (PR), and biological studies have reported that the PR expression is increased in GC,^[36]^ with elevated PR expression being associated with poorer prognosis in GC.^[37]^ These findings are consistent with our results, where higher progesterone concentrations were associated with an increased risk of high-grade lesions or GC. Moreover, this evidence further supports the positive association between pregnenolone levels and incident GC risk observed during the 11.8-year prospective follow-up. As illustrated in the sex steroid hormone metabolism pathway,^[32]^ pregnenolone can be metabolized into progesterone, thereby influencing the development of GC. In addition, higher levels of 17α-hydroxypregnenolone, which acts as a metabolite of pregnenolone, were also associated with an increased risk of high-grade lesions or GC. Interestingly, the associations of these sex hormone metabolites with GC and precancerous lesions remained consistent across subgroup analyses stratified by *H. pylori* status. Therefore, further mechanistic studies are needed to elucidate the complex effects of sex hormone metabolites on GC and precancerous lesions, as well as the potential interactive effects of *H. pylori* infection on these associations.

In the prospective analysis, we observed an increased risk of incident GC in participants with higher epitestosterone levels. To our knowledge, the biosynthetic and metabolic pathways of epitestosterone in humans are not fully elucidated, and studies exploring the association between epitestosterone and GC are limited. However, two Mendelian randomization analyses have reported that testosterone is associated with a lower risk of GC in men.^[21,38]^ Epitestosterone, a natural steroid metabolite and the 17 alpha-hydroxy epimer of testosterone produced by the immature testis, may be involved in regulating androgen-dependent events such as prostate growth.^[39]^ It may also function as a 5 alpha-reductase inhibitor and an antiandrogen by competitively binding to the AR, thereby inhibiting testosterone biosynthesis.^[40]^ These findings imply that epitestosterone may contribute to the development of GC by affecting the metabolism pathway of testosterone. However, we did not observe a significant association between epitestosterone and GC risk in subgroup analyses, which suggests that *H. pylori* infection may interact with epitestosterone levels, potentially modifying its association with GC risk.

In addition, we observed a positive association between higher levels of SHBG and incident GC in men. This finding is consistent with the results of two prospective studies based on the UK Biobank, which reported that elevated SHBG levels were associated with an increased risk of GC.^[23,41]^ Another meta-analysis also showed that higher levels of SHBG increased the risk of GC in men.^[42]^ Our study provides novel epidemiological evidence supporting the hypothesis that sex hormone dysregulation may play a role in gastric carcinogenesis.

This study has several strengths. We collected extensive information on potential confounders of GC, including common risk factors such as age, BMI, smoking status, alcohol consumption, *etc*., as well as the established serologic biomarkers such as *H. pylori* serostatus in the current screening guideline of Chinese populations. By adjusting for key risk factors and stratifying by *H. pylori* infection, we investigated the associations of up to 20 sex steroid hormone metabolites with GC and its precursors, thereby providing additional biomarkers for identifying high-risk populations for GC. To our knowledge, this is the first study to simultaneously investigate the associations between sex hormones and GC and precancerous lesions (high-grade lesions and IM) in Chinese men, offering insight into the potential role of sex hormones in the development of GC. However, this study also had several limitations. The modest sample size may have limited our ability to identify significant associations between certain sex steroid hormone metabolites and GC and its precursors; meanwhile, the limited number of GC cases constrained the performance of subsite analysis of cardia and non-cardia GC.

In conclusion, we found that several sex steroid hormone metabolites were associated with GC and precancerous lesions in a prospective cohort of Chinese men. These findings suggest a role for sex hormones in the carcinogenesis of GC. With further validation, these findings may contribute to the understanding of the etiology of GC, and help provide novel biomarkers for the precision identification of high-risk populations and risk prediction of GC.

## Supplementary Information

Supplementary materials are only available at the official site of the journal (www.intern-med.com).

## Supplementary Material

Supplementary Material Details
