# Supplementary material for: Associations between serum sex steroid hormone metabolites and gastric cancer and precancerous lesions in men: A 11.8-year prospective study
Source: J Transl Int Med. 2025 Oct 16;13(5):436–55. doi: 10.1515/jtim-2025-0041 (PMC12569587; doi:10.1515/jtim-2025-0041)
Supplement: Supplementary file 1 — Supplementary Material Details [file jtim-2025-0041_sm.pdf]

Supplementary Materials

Associations between serum sex steroid hormone metabolites and gastric cancer and precancerous lesions in men: a 11.8-year prospective study

Jiayue Li, Wei Cui, Feifan He, Zhiyuan Fan, Liyan Xue, Wei Rao, Zongdan Wang, Zeming Wu, Jianhua Gu, Xinqing Li, Wenqiang Wei*, Shaoming Wang*

Technical information

Liquid chromatograph mass spectrometer (LC-MS) grade methanol, ammonium hydroxide and liquid chromatography (LC) grade dichloromethane were purchased from Fisher Scientific (Fair Lawn, USA), and the ultra-pure water used in the study was prepared using an in-house Milli-Q purification system (Merck Millipore, USA). Unlabelled pure standards including testosterone, 16α-hydroxytestosterone, androstenedione , 17α-hydroxyprogesterone, 17α-hydroxypregnenolone, progesterone, estrone, epitestosterone, dehydroepiandrosterone (DHEA), pregnenolone, dihydrotestosterone, etiocholanolone, 17-epiestriol, estradiol, 2-methoxyestrone, 2-methoxyestradiol, 16-epiestriol, androsterone, 11-oxoetiocholanolone, and 6β-hydroxytestosterone were purchased from Sigma-Aldrich (Merck KGaA, Darmstadt, Germany). Deuterium isotope-labelled internal standards including progesterone-d9, testosterone-d3, 17-hydroxyprogesterone-d8, cortisol-d4, dihydrotestosterone-d3, dehydroepiandrosterone-d6, pregnenolone-d4, and estrone-d4 were supplied by Toronto Research Chemicals Inc. (Toronto, Canada) and Cambridge Isotope Laboratories (Andover, USA). The steroid enrichment sample was prepared

using a previously reported modified solid phase extraction (SPE) protocol[1].

Steroid metabolites were quantified using a liquid chromatography-tandem mass spectrometry (LC-MS/MS) system consisting of a Nexera 20AD ultra-high performance liquid chromatograph (Shimadzu Corporation, Japan) and an AB Sciex 5500 QTRAP equipped with a TurboV electrospray ionization source (SCIEX, Singapore). Metabolites were initially separated on an Acquity BEH C18 column (1.7 μm, 2.1×100 mm, Waters Corporation, USA) using binary mobile phases consisting of water (A) and methanol (B), both containing 0.02% ammonium hydroxide, with a linear gradient as follows: 40% B initially, increasing to 100% in 8 min, maintained until 9.5 min, then decreasing to 40% at 9.6 min and equilibrating until 13 min. The flow rate, column temperature and injection volume were set to 0.3 mL/min, 50°C and 20 μL, respectively. The selected ion monitoring scan mode was used for metabolites detection, with detailed parameters listed in the **Supplementary Table S1**. The main ion source settings were optimized as follows: ionization voltage 5.5 kV (positive mode) and 4.5 kV (negative mode), source temperature 550 °C, curtain gas at 35 psi and GS at 45 psi. Data were acquired using an Analyst workstation (SCIEX, Singapore) and then processed using MultiQuant software (SCIEX, Singapore), with internal standard calibration for steroid metabolites quantification. In addition, several quality control (QC) strategies were employed to evaluate and ensure the analytical performance during batch processing. First, QC samples were prepared by evenly mixing aliquots of randomly assigned samples and were inserted into the analytical

sequence for each sample, which was completely randomized. Finally, a total of 35 QC samples were evenly distributed on each SPE plate and analyzed by LC-MS/MS. The analytical performance, including reproducibility, *e.g.* the coefficient of variance of the concentration determinations in these QC samples, and sensitivity of the LC-MS/MS method (limit of detection, limit of quantification) were reported in the **Supplementary Table S2-3**.

References

1. Koal T, Schmiederer D, Pham-Tuan H, Röhring C, Rauh M. Standardized LC-MS/MS based steroid hormone profile-analysis. J Steroid Biochem Mol Biol. 2012;129(3-5):129-138. doi: 10.1016/j.jsbmb.2011.12.001.

		IM participants vs Normal participants			High-grade lesions or GC participants vs Normal participants		
**Sex hormone** metabolites	***N*=140**	***N*=51**	**OR (95% CI)***	***P* for trend**	***N*=13**	**OR (95% CI)***	***P* for trend**
Sex hormone binding globulin (ng/L)				0.70			0.30
<51.56	39	12	Reference		4	Reference	
51.56–<69.14	31	15	1.56 (0.61 , 3.97)		1	0.24 (0.02 , 2.45)	
69.14–<94.54	38	8	0.72 (0.25 , 2.06)		5	0.78 (0.16 , 3.70)	
≥94.54	32	16	1.55 (0.57 , 4.20)		3	0.22 (0.03 , 1.78)	
Continuous(log)			1.38 (0.63 , 3.04)			0.47 (0.10 , 2.13)	
Testosterone (ng/L)				0.72			0.55
<5096.25	35	12	Reference		5	Reference	
5096.25–<6339.90	38	12	0.79 (0.30 , 2.08)		0	0.00 (0.00 , 0.00)	
6339.90–<7807.03	30	13	1.23 (0.47 , 3.23)		4	0.64 (0.13 , 3.10)	
≥7807.03	37	14	1.03 (0.39 , 2.74)		4	0.31 (0.06 , 1.72)	
Continuous(log)			1.55 (0.53 , 4.49)			0.19 (0.05 , 0.63)	
16a–Hydroxytestosterone (ng/L)				0.92			0.96
<1107.14	28	8	Reference		4	Reference	
1107.14–<1456.78	37	17	1.59 (0.57 , 4.42)		2	0.28 (0.04 , 1.90)	
1456.78–< 1871.11	42	15	1.16 (0.42 , 3.19)		2	0.25 (0.04 , 1.67)	
≥1871.11	33	11	1.14 (0.39 , 3.39)		5	0.80 (0.15 , 4.24)	
Continuous(log)			1.09 (0.44 , 2.71)			1.02 (0.22 , 4.71)	
Androstenedione (ng/L)				0.98			0.09
<945.09	31	6	Reference		1	Reference	
945.09–<1252.99	32	20	4.06 (1.33 , 12.40)		3	4.40 (0.40 , 48.88)	
1252.99–<1613.45	38	13	2.04 (0.64 , 6.48)		0	0.00 (0.00 , 0.00)	
≥1613.45	39	12	1.82 (0.55 , 5.98)		9	8.08 (0.82 , 79.56)	
Continuous(log)			1.18 (0.54 , 2.57)			4.84 (1.17 , 20.01)	
17a–Hydroxyprogesterone (ng/L)				0.15			0.42
<781.64	35	15	Reference		2	Reference	
781.64–<994.02	38	16	1.04 (0.43 , 2.49)		2	0.97 (0.12 , 7.73)	
994.02–<1252.98	26	10	0.89 (0.34 , 2.33)		4	2.20 (0.34 , 14.15)	
≥1252.98	41	10	0.50 (0.19 , 1.28)		5	1.83 (0.29 , 11.42)	
Continuous(log)			0.53 (0.22 , 1.28)			1.76 (0.34 , 9.12)	
17a–Hydroxypregnenolone (ng/L)				0.18			0.02
<2438.82	27	13	Reference		2	Reference	
2438.82–<3489.98	32	15	1.20 (0.46 , 3.08)		0	0.00 (0.00 , 0.00)	
3489.98–<5041.80	40	13	0.85 (0.32 , 2.24)		2	0.93 (0.11 , 8.02)	
≥5041.80	41	10	0.55 (0.20 , 1.52)		9	5.38 (0.90 , 32.24)	
Continuous(log)			0.51 (0.25 , 1.04)			5.99 (1.55 , 23.18)	
Progesterone (ng/L)				> 0.05			0.09
<47.40	31	17	Reference		2	Reference	
47.40–<59.22	38	17	0.66 (0.28 , 1.58)		2	0.74 (0.09 , 6.26)	
59.22–<73.86	34	5	0.24 (0.08 , 0.75)		2	0.76 (0.08 , 6.77)	
≥73.86	37	12	0.50 (0.19 , 1.27)		7	4.15 (0.60 , 28.93)	
Continuous(log)			0.49 (0.20 , 1.21)			6.91 (1.61 , 29.59)	
Estrone (ng/L)				0.90			0.04
<53.37	31	14	Reference		2	Reference	
53.37–<63.53	32	11	0.75 (0.28 , 1.98)		1	0.75 (0.06 , 9.91)	
63.53–<75.23	40	9	0.51 (0.19 , 1.35)		1	0.40 (0.03 , 4.95)	
≥75.23	37	17	1.02 (0.42 , 2.44)		9	5.74 (0.89 , 37.18)	
Continuous(log)			1.08 (0.33 , 3.53)			5.22 (0.51 , 53.79)	
Epitestosterone (ng/L)				0.81			0.33
<68.61	35	9	Reference		4	Reference	
68.61–<108.79	34	17	2.19 (0.82 , 5.87)		3	0.51 (0.09 , 2.95)	
108.79–<151.88	38	14	1.47 (0.54 , 4.02)		1	0.12 (0.01 , 1.28)	
≥151.88	33	11	1.42 (0.48 , 4.23)		5	0.48 (0.08 , 2.81)	
Continuous(log)			0.98 (0.52 , 1.85)			0.70 (0.23 , 2.18)	
Dehydroepiandrosterone (ng/L)				0.59			0.21
<990.72	30	14	Reference		1	Reference	
990.72–<1445.69	29	13	1.34 (0.51 , 3.56)		3	5.83 (0.45 , 76.04)	
1445.69–<2137.65	42	10	0.63 (0.24 , 1.67)		6	7.86 (0.75 , 82.38)	
≥2137.65	39	14	0.98 (0.37 , 2.61)		3	4.92 (0.40 , 60.16)	
Continuous(log)			0.93 (0.50 , 1.74)			2.01 (0.59 , 6.86)	
Pregnenolone (ng/L)				0.64			0.38
<158.98	27	9	Reference		3	Reference	
158.98–<519.01	34	14	1.46 (0.52 , 4.15)		3	1.57 (0.24 , 10.30)	
519.01–<788.31	34	11	1.17 (0.40 , 3.40)		1	0.34 (0.03 , 4.15)	
≥788.31	45	17	1.43 (0.52 , 3.93)		6	2.76 (0.48 , 15.91)	
Continuous(log)			1.03 (0.89 , 1.20)			1.36 (0.89 , 2.08)	
Dihydrotestosterone (ng/L)				0.69			<0.05
<442.93	33	11	Reference		5	Reference	
442.93–<559.25	30	11	0.88 (0.32 , 2.42)		3	0.45 (0.08 , 2.43)	
559.25–<761.73	33	17	1.39 (0.55 , 3.51)		1	0.12 (0.01 , 1.26)	
≥761.73	44	12	0.70 (0.26 , 1.90)		4	0.19 (0.03 , 1.11)	
Continuous(log)			0.94 (0.41 , 2.17)			0.17 (0.04 , 0.63)	
Etiocholanolone (ng/L)				0.31			0.11
<151.56	30	10	Reference		2	Reference	
151.56 –<200.68	38	11	1.02 (0.36 , 2.83)		2	0.88 (0.10 , 7.55)	
200.68–<264.40	33	13	1.41 (0.51 , 3.88)		4	2.16 (0.32 , 14.71)	
≥264.40	39	17	1.52 (0.57 , 4.06)		5	3.40 (0.51 , 22.61)	
Continuous(log)			1.14 (0.45 , 2.92)			2.14 (0.35 , 12.93)	
17–Epiestriol (ng/L)				0.45			0.53
<11.47	34	18	Reference		6	Reference	
11.47–<14.92	33	7	0.43 (0.15 , 1.18)		2	0.37 (0.06 , 2.13)	
14.92–<19.26	34	15	0.86 (0.36 , 2.02)		2	0.37 (0.07 , 2.06)	

≥19.26	39	11	0.59 (0.24 , 1.46)		3	0.70 (0.14 , 3.46)	
Continuous(log)			0.71 (0.39 , 1.29)			0.58 (0.16 , 2.09)	
Estradiol (ng/L)				0.60			0.09
<25.25	35	12	Reference		4	Reference	
25.25–<36.94	31	8	0.72 (0.25 , 2.02)		6	1.73 (0.40 , 7.58)	
36.94–<47.21	35	14	1.06 (0.41 , 2.70)		2	0.39 (0.06 , 2.45)	
≥47.21	39	17	1.15 (0.47 , 2.81)		1	0.23 (0.02 , 2.27)	
Continuous(log)			1.02 (0.71 , 1.48)			0.73 (0.44 , 1.20)	
2–Methoxyestrone (ng/L)				0.87			0.76
<13.57	39	14	Reference		3	Reference	
13.57 –<18.03	37	15	1.17 (0.48 , 2.85)		4	1.64 (0.31 , 8.62)	
18.03–<23.67	35	11	0.80 (0.31 , 2.05)		4	1.36 (0.26 , 7.18)	
≥23.67	29	11	1.06 (0.41 , 2.70)		2	0.69 (0.09 , 5.28)	
Continuous(log)			0.98 (0.49 , 1.97)			0.82 (0.22 , 3.02)	
2–Methoxyestradiol (ng/L)				0.19			0.16
<9.17	28	12	Reference		6	Reference	
9.17–<12.70	39	19	1.08 (0.44 , 2.66)		2	0.19 (0.03 , 1.13)	
12.70 –<19.22	35	9	0.61 (0.22 , 1.70)		3	0.38 (0.07 , 1.94)	
≥19.22	38	11	0.61 (0.22 , 1.68)		2	0.23 (0.04 , 1.44)	
Continuous(log)			0.86 (0.49 , 1.50)			0.42 (0.17 , 1.05)	
16–Epiestriol (ng/L)				0.95			0.93
<20.92	35	11	Reference		4	Reference	
20.92–<37.08	33	14	1.18 (0.45 , 3.14)		2	0.40 (0.06 , 2.61)	
37.08 <54.84	36	15	1.33 (0.53 , 3.39)		3	0.56 (0.10 , 3.06)	
≥54.84	36	11	0.93 (0.35 , 2.48)		4	0.82 (0.16 , 4.18)	
Continuous(log)			1.02 (0.73 , 1.42)			1.07 (0.58 , 1.96)	
Androsterone (ng/L)				0.96			0.20
<266.25	36	12	Reference		2	Reference	
266.25–<356.07	37	15	1.55 (0.61 , 3.94)		4	2.36 (0.37 , 15.05)	
356.07–<462.57	36	15	1.28 (0.50 , 3.31)		2	0.75 (0.09 , 6.52)	
≥462.57	31	9	1.02 (0.35 , 2.94)		5	4.71 (0.73 , 30.59)	
Continuous(log)			1.09 (0.55 , 2.19)			4.18 (0.97 , 17.94)	
11–Oxoetiocholanolone (ng/L)				0.49			0.88
<26.12	31	10	Reference		2	Reference	
26.12–<58.21	29	14	1.70 (0.62 , 4.64)		4	1.83 (0.29 , 11.62)	
58.21 –<142.89	39	17	1.43 (0.55 , 3.70)		4	1.17 (0.18 , 7.60)	
≥142.89	41	10	0.76 (0.27 , 2.13)		3	1.09 (0.16 , 7.33)	
Continuous(log)			0.97 (0.77 , 1.23)			0.98 (0.64 , 1.50)	
6ß–Hydroxytestosterone (ng/L)				0.21			0.75
<33.77	28	16	Reference		5	Reference	
33.77–<65.56	35	9	0.41 (0.15 , 1.13)		0	0.00 (0.00 , 0.00)	
65.56–<136.71	34	14	0.71 (0.28 , 1.77)		4	0.65 (0.15 , 2.89)	
≥136.71	43	12	0.47 (0.19 , 1.16)		4	0.55 (0.12 , 2.42)	
Continuous(log)			0.80 (0.59 , 1.09)			0.80 (0.43 , 1.47)	
Parent estrogens (ng/L)				0.80			0.21
<82.39	35	13	Reference		3	Reference	
82.39–<101.52	30	10	0.92 (0.34 , 2.51)		0	0.00 (0.00 , 0.00)	
101.52–<118.98	34	9	0.72 (0.27 , 1.96)		4	1.15 (0.21 , 6.41)	
≥118.98	41	19	1.16 (0.48 , 2.76)		6	1.89 (0.39 , 9.23)	
Continuous(log)			1.08 (0.38 , 3.09)			1.02 (0.14 , 7.45)	
Testosterone: parent estrogens ratio				0.31			0.57
<50.80	37	14	Reference		4	Reference	
50.80–<62.20	35	6	0.52 (0.17 , 1.55)		2	0.49 (0.08 , 3.03)	
62.20–<81.57	34	15	1.26 (0.50 , 3.14)		3	0.59 (0.11 , 3.09)	
≥81.57	34	16	1.32 (0.53 , 3.32)		4	0.58 (0.10 , 3.24)	
Continuous(log)			1.29 (0.53 , 3.18)			0.21 (0.06 , 0.67)	
Testosterone: estradiol ratio				0.95			0.36
<131.65	36	15	Reference		1	Reference	
131.65–<176.29	34	10	0.77 (0.29 , 2.02)		4	4.63 (0.44 , 48.45)	
176.29–<250.66	35	12	0.83 (0.33 , 2.10)		3	2.72 (0.25 , 29.79)	
≥250.66	35	14	1.02 (0.41 , 2.54)		5	4.40 (0.43 , 45.56)	
Continuous(log)			1.03 (0.72 , 1.46)			0.98 (0.51 , 1.88)	
Androstenedione: estrone ratio				0.51			0.19
<15.49	30	11	Reference		2	Reference	
15.49–<19.12	36	7	0.55 (0.18 , 1.70)		1	0.40 (0.03 , 5.06)	
19.12–<24.69	35	19	1.50 (0.57 , 3.95)		3	1.12 (0.16 , 8.04)	
≥24.69	39	14	1.01 (0.38 , 2.69)		7	2.13 (0.35 , 13.11)	
Continuous(log)			1.20 (0.47 , 3.06)			3.84 (0.74 , 19.85)	

OR axis (left): 0.1 0.2 0.5 1 2 5 10 — Low risk ← → High risk

OR axis (right): 0.01 0.05 0.2 1 5 20 100 — Low risk ← → High risk

SP Figure 1. Associations between sex hormone metabolites and gastric cancer and its precursors among *H. pylori* CagA-negative participants.

***: Adjusted for age, body mass index, smoking, drinking, history of upper gastrointestinal disease, family history of cancer.**

Abbreviations: IM, intestinal metaplasia; GC, gastric cancer

		Gastric cancer participants vs Control participants		
**Sex hormone** metabolites	***N*=176**	***N*=15**	**HR (95% CI)***	***P* for trend**
Sex hormone binding globulin (ng/L)				0.02
<51.56	50	1	Reference	
51.56–<69.14	44	2	2.92 (0.24 , 35.39)	
69.14–<94.54	40	6	6.39 (0.70 , 58.16)	
≥94.54	42	6	11.15 (1.05 , 118.70)	
Continuous(log)			4.77 (1.18 , 19.34)	
Testosterone (ng/L)				0.10
<5096.25	44	3	Reference	
5096.25–<6339.90	47	3	1.54 (0.29 , 8.33)	
6339.90–<7807.03	40	3	1.69 (0.28 , 10.16)	
≥7807.03	45	6	3.57 (0.74 , 17.27)	
Continuous(log)			3.65 (0.64 , 20.94)	
16a–Hydroxytestosterone (ng/L)				0.01
<1107.14	34	2	Reference	
1107.14–<1456.78	53	1	0.29 (0.03 , 3.23)	
1456.78–< 1871.11	50	7	4.05 (0.79 , 20.66)	
≥1871.11	39	5	4.01 (0.73 , 22.17)	
Continuous(log)			4.16 (0.95 , 18.18)	
Androstenedione (ng/L)				0.12
<945.09	37	0		
945.09–<1252.99	45	7	Reference	
1252.99–<1613.45	49	2	0.34 (0.07 , 1.73)	
≥1613.45	45	6	0.98 (0.32 , 3.05)	
Continuous(log)			2.51 (0.73 , 8.65)	
17a–Hydroxyprogesterone (ng/L)				0.84
<781.64	46	4	Reference	
781.64–<994.02	51	3	0.86 (0.18 , 4.03)	
994.02–<1252.98	33	3	1.12 (0.24 , 5.17)	
≥1252.98	46	5	1.09 (0.28 , 4.27)	
Continuous(log)			0.77 (0.21 , 2.82)	
17a–Hydroxypregnenolone (ng/L)				0.38
<2438.82	38	2	Reference	
2438.82–<3489.98	43	4	2.40 (0.41 , 14.00)	
3489.98–<5041.80	48	5	2.26 (0.42 , 12.27)	
≥5041.80	47	4	2.35 (0.42 , 13.31)	
Continuous(log)			1.06 (0.40 , 2.80)	
Progesterone (ng/L)				0.49
<47.40	45	3	Reference	
47.40–<59.22	52	3	0.90 (0.17 , 4.92)	
59.22–<73.86	33	6	3.19 (0.74 , 13.64)	
≥73.86	46	3	1.01 (0.18 , 5.55)	
Continuous(log)			0.99 (0.35 , 2.80)	
Estrone (ng/L)				0.55
<53.37	42	3	Reference	
53.37–<63.53	41	2	0.40 (0.06 , 2.63)	
63.53–<75.23	44	5	1.41 (0.33 , 6.07)	
≥75.23	49	5	1.04 (0.23 , 4.68)	
Continuous(log)			2.47 (0.26 , 23.17)	
Epitestosterone (ng/L)				0.19
<68.61	44	0		
68.61–<108.79	45	6	Reference	
108.79–<151.88	48	4	0.71 (0.19 , 2.60)	
≥151.88	39	5	0.87 (0.23 , 3.26)	
Continuous(log)			2.46 (0.84 , 7.20)	
Dehydroepiandrosterone (ng/L)				0.40
<990.72	42	2	Reference	
990.72–<1445.69	37	5	3.28 (0.58 , 18.48)	
1445.69–<2137.65	47	5	2.95 (0.55 , 15.67)	
≥2137.65	50	3	2.35 (0.36 , 15.36)	
Continuous(log)			1.23 (0.44 , 3.39)	
Pregnenolone (ng/L)				0.01
<158.98	35	1	Reference	
158.98–<519.01	45	3	2.94 (0.28 , 30.67)	

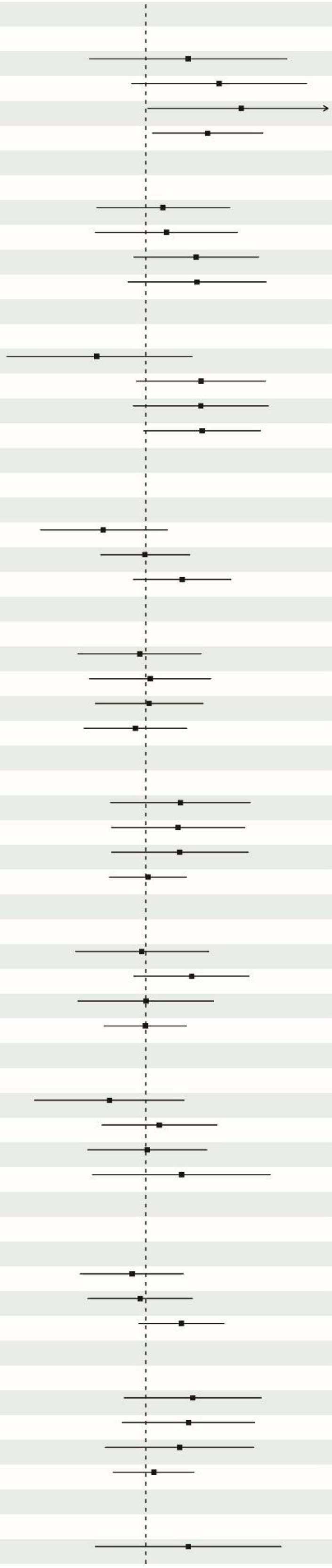

519.01–<788.31	40	5	9.06 (1.01 , 81.40)	
≥788.31	56	6	11.34 (1.27 , 101.59)	
Continuous(log)			1.74 (1.01 , 3.01)	
Dihydrotestosterone (ng/L)				0.03
<442.93	43	1	Reference	
442.93–<559.25	40	1	1.28 (0.08 , 21.11)	
559.25–<761.73	43	7	8.29 (0.89 , 77.66)	
≥761.73	50	6	7.67 (0.81 , 72.57)	
Continuous(log)			4.93 (1.14 , 21.33)	
Etiocholanolone (ng/L)				0.06
<151.56	37	3	Reference	
151.56 –<200.68	47	2	0.50 (0.08 , 3.19)	
200.68–<264.40	41	5	2.78 (0.60 , 12.95)	
≥264.40	51	5	3.45 (0.66 , 17.86)	
Continuous(log)			3.06 (0.64 , 14.68)	
17–Epiestriol (ng/L)				0.47
<11.47	48	4	Reference	
11.47–<14.92	39	1	0.31 (0.03 , 2.86)	
14.92–<19.26	42	7	3.80 (0.92 , 15.80)	
≥19.26	47	3	0.82 (0.18 , 3.84)	
Continuous(log)			0.99 (0.35 , 2.79)	
Estradiol (ng/L)				0.86
<25.25	43	4	Reference	
25.25–<36.94	35	4	0.71 (0.15 , 3.39)	
36.94–<47.21	46	3	0.64 (0.13 , 3.24)	
≥47.21	52	4	0.86 (0.20 , 3.77)	
Continuous(log)			0.87 (0.46 , 1.63)	
2–Methoxyestrone (ng/L)				0.48
<13.57	48	5	Reference	
13.57 –<18.03	49	3	0.59 (0.13 , 2.62)	
18.03–<23.67	44	2	0.24 (0.04 , 1.36)	
≥23.67	35	5	0.68 (0.18 , 2.65)	
Continuous(log)			0.68 (0.21 , 2.15)	
2–Methoxyestradiol (ng/L)				0.49
<9.17	35	5	Reference	
9.17–<12.70	55	3	0.39 (0.09 , 1.73)	
12.70 –<19.22	38	6	1.30 (0.32 , 5.30)	
≥19.22	48	1	0.24 (0.03 , 2.20)	
Continuous(log)			0.82 (0.35 , 1.95)	
16–Epiestriol (ng/L)				0.38
<20.92	42	4	Reference	
20.92–<37.08	41	6	2.38 (0.51 , 11.11)	
37.08 <54.84	49	2	0.45 (0.07 , 2.71)	
≥54.84	44	3	0.94 (0.19 , 4.79)	
Continuous(log)			0.96 (0.61 , 1.50)	
Androsterone (ng/L)				0.10
<266.25	46	2	Reference	
266.25–<356.07	49	3	1.50 (0.23 , 9.68)	
356.07–<462.57	47	4	1.51 (0.25 , 9.24)	
≥462.57	34	6	4.28 (0.71 , 25.75)	
Continuous(log)			2.93 (0.71 , 12.18)	
11–Oxoetiocholanolone (ng/L)				0.14
<26.12	38	3	Reference	
26.12–<58.21	41	2	0.41 (0.07 , 2.58)	
58.21 –<142.89	51	5	1.39 (0.31 , 6.29)	
≥142.89	46	5	2.35 (0.47 , 11.83)	
Continuous(log)			1.43 (0.84 , 2.44)	
6ß–Hydroxytestosterone (ng/L)				0.87
<33.77	40	4	Reference	
33.77–<65.56	41	3	0.77 (0.16 , 3.72)	
65.56–<136.71	43	5	1.94 (0.41 , 9.15)	
≥136.71	52	3	0.70 (0.15 , 3.38)	
Continuous(log)			0.94 (0.57 , 1.56)	
Parent estrogens (ng/L)				0.44
<82.39	45	3	Reference	

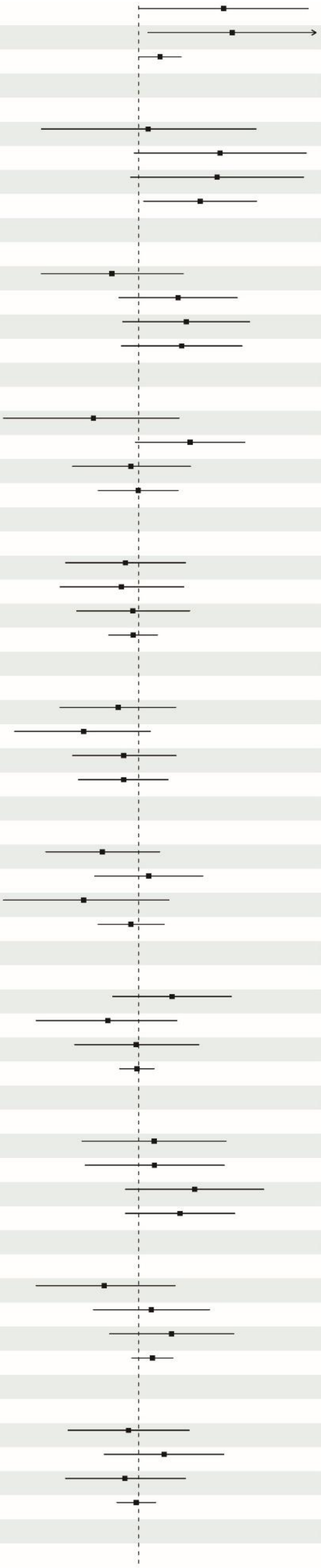

82.39–<101.52	37	3	1.25 (0.24 , 6.50)	
101.52–<118.98	40	3	0.67 (0.13 , 3.60)	
≥118.98	54	6	2.01 (0.47 , 8.53)	
Continuous(log)			1.38 (0.19 , 10.06)	
Testosterone: parent estrogens ratio				0.24
<50.80	48	3	Reference	
50.80–<62.20	39	2	1.03 (0.16 , 6.66)	
62.20–<81.57	42	7	2.98 (0.69 , 12.93)	
≥81.57	47	3	1.80 (0.30 , 10.93)	
Continuous(log)			2.50 (0.48 , 12.92)	
Testosterone: estradiol ratio				0.32
<131.65	48	3	Reference	
131.65–<176.29	41	3	1.22 (0.24 , 6.23)	
176.29–<250.66	42	5	2.26 (0.49 , 10.44)	
≥250.66	45	4	2.08 (0.40 , 10.78)	
Continuous(log)			1.32 (0.77 , 2.27)	
Androstenedione: estrone ratio				0.16
<15.49	38	3	Reference	
15.49–<19.12	42	1	0.39 (0.04 , 3.94)	
19.12–<24.69	47	7	2.91 (0.72 , 11.84)	
≥24.69	49	4	1.84 (0.38 , 8.98)	
Continuous(log)			2.25 (0.57 , 8.88)	

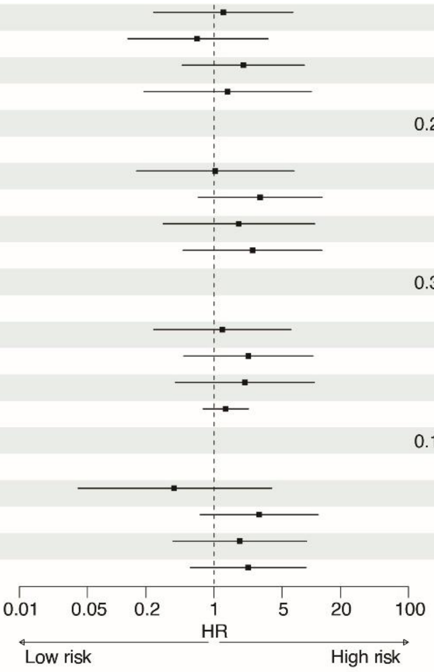

SP Figure 2. Associations between baseline sex hormone metabolites and incident gastric cancer risk among *H. pylori* CagA-negative participants.
*: Adjusted for age, body mass index, smoking, drinking, history of upper gastrointestinal disease, family history of cancer.

Supplementary Table S1: LC-MS/MS detection parameters for steroid hormones quantitation

Type	Name	Internal standards assigned	SRM transition (Q1/Q3)	Retention time/min	DP Voltage/V	CE Voltage/V	ESI Polarity
Sex Steroid hormone metabolites	Testosterone	Testosterone-d3	289.2/97.1	5.49	155	30	ESI(+)
	16α-Hydroxytestosterone	Cortisol-d4	305.2/97.1	4.02	160	30	ESI(+)
	Androstenedione	Testosterone-d3	287.2/97.1	5.22	165	29	ESI(+)
	17α-Hydroxyprogesterone	17-Hydroxyprogesterone-d8	331.2/97.1	5.63	166	33	ESI(+)
	17α-Hydroxypregnenolone	Estrone-d4	331.2/287.3	5.63	-120	-27	ESI(-)
	Progesterone	Progesterone-d9	315.2/97.1	6.46	155	27	ESI(+)
	Estrone	Estrone-d4	269.2/145.1	5.19	-220	-52	ESI(-)
	Epitestosterone	Dihydrotestosterone-d3	289.2/97.1	6.01	160	31	ESI(+)
	Dehydroepiandrosterone	Dehydroepiandrosterone-d6	289.2/253.2	5.50	180	17	ESI(+)
	Pregnenolone	Pregnenolone-d4	317.2/299.2	6.84	200	12	ESI(+)
	Dihydrotestosterone	Dihydrotestosterone-d3	291.2/255.1	6.11	185	21	ESI(+)
	Etiocholanolone	Progesterone-d9	291.2/255.2	6.59	160	19	ESI(+)
	17-Epiestriol	Estrone-d4	287.2/145.1	3.99	-180	-53	ESI(-)
	Estradiol	Estrone-d4	271.2/145.1	5.12	-200	-55	ESI(-)
	2-Methoxyestrone	Estrone-d4	299.2/284.2	5.45	-180	-32	ESI(-)
	2-Methoxyestradiol	Estrone-d4	301.2/286.2	5.48	-180	-30	ESI(-)
	16-Epiestriol	Estrone-d4	287.2/171.1	3.81	-180	-52	ESI(-)

	Androsterone	Progesterone-d9	291.2/273.2	6.59	180	14	ESI(+)
	11-Oxoetiocholanolone	Estrone-d4	303.2/285.1	5.10	-180	-38	ESI(-)
	6β-Hydroxytestosterone	Cortisol-d4	305.2/269.2	3.42	160	21	ESI(+)
Internal standards	Progesterone-d9		324.3/100.1	6.42	155	35	ESI(+)
	Testosterone-d3		292.2/97.1	5.47	155	34	ESI(+)
	17-Hydroxyprogesterone-d8		339.3/100.1	5.62	166	40	ESI(+)
	Cortisol-d4		367.2/121.1	3.98	156	22	ESI(+)
	Dihydrotestosterone-d3		294.3/258.1	6.09	185	23	ESI(+)
	Dehydroepiandrosterone-d6		295.2/259.2	5.69	180	14	ESI(+)
	Pregnenolone-d4		321.3/303.3	6.81	200	17	ESI(+)
	Estrone-d4		273.2/147.1	5.17	-220	-52	ESI(-)

LC-MS/MS: liquid chromatography-tandem mass spectrometry; SRM: selected reaction monitoring; DP: declustering potential; CE: collision energy; ESI: electrospray ionization

Supplementary Table S2: CV% of concentration determination values in all QC samples

Name	CV% in QC samples
Testosterone	1.14
16α-Hydroxytestosterone	1.50
Androstenedione	1.96
17α-Hydroxyprogesterone	2.02
17α-Hydroxypregnenolone	2.40
Progesterone	2.87
Estrone	5.42
Epitestosterone	9.59
Dehydroepiandrosterone	10.88
Pregnenolone	10.95
Dihydrotestosterone	12.19
Etiocholanolone	15.13
17-Epiestriol	19.80
Estradiol	21.22
2-Methoxyestrone	23.36
2-Methoxyestradiol	26.14
16-Epiestriol	26.62
Androsterone	28.80
11-Oxoetiocholanolone	44.20
6β-Hydroxytestosterone	45.98

CV, coefficients of variation; QC: quality control

Supplementary Table S3: Sensitivity and Linerity range of the LC-MS/MS method

Name	LOD (ng/mL)	LOQ (ng/mL)	Linerity (ng/mL)
Testosterone	0.25	0.25	0.25~200
16α-Hydroxytestosterone	2.5	2.5	2.5~200
Androstenedione	0.25	2.5	2.5~200
17α-Hydroxyprogesterone	0.25	5	5~200
17α-Hydroxypregnenolone	0.05	0.5	0.5~200
Progesterone	0.25	0.25	0.25~200
Estrone	0.05	0.05	0.05~100
Epitestosterone	0.5	2.5	2.5~200
Dehydroepiandrosterone	0.5	0.5	0.5~200
Pregnenolone	5	5	5~200
Dihydrotestosterone	5	5	5~200
Etiocholanolone	0.5	2.5	2.5~200
17-Epiestriol	0.05	0.05	0.05~200
Estradiol	0.05	0.05	0.05~200
2-Methoxyestrone	0.05	0.25	0.25~100
2-Methoxyestradiol	0.05	0.05	0.05~200
16-Epiestriol	0.05	0.05	0.05~200
Androsterone	2.5	2.5	2.5~200
11-Oxoetiocholanolone	0.5	2.5	2.5~200
6β-Hydroxytestosterone	2.5	2.5	2.5~200

LC-MS/MS: liquid chromatography-tandem mass spectrometry; LOD, limit of detection; LOQ, limit of quantification.
